# Maladaptive Eating Behaviors and Childhood Trauma: A Focus on Food Addiction

**DOI:** 10.7759/cureus.26966

**Published:** 2022-07-18

**Authors:** Maxime Legendre, Stéphane Sabourin, Catherine Bégin

**Affiliations:** 1 School of Psychology, Laval University, Quebec City, CAN

**Keywords:** bullying, interpersonal trauma, peer victimization, obesity, food addiction, binge eating, abuse

## Abstract

Background

Many studies have highlighted the clinical relevance of food addiction (FA) by showing its association with psychopathological severity and even more when co-occurring with binge eating disorder. It was suggested that the association between FA and greater psychopathological severity could be accounted for by a history of traumatic experiences. The present study examined the relationship between childhood trauma (including peer victimization, abuse, and neglect) and maladaptive eating behaviors (FA, binge eating, and grazing) and explored whether childhood trauma predicts FA when controlling for binge eating, grazing, and other confounding variables.

Methods

One hundred fourteen adult women seeking psychological help for problems related to eating or weight completed questionnaires measuring FA, binge eating, grazing, depressive symptoms, peer victimization, and childhood abuse and neglect.

Results

FA showed significantly small to moderate positive correlations with all measures of childhood trauma, except for physical neglect. A hierarchical regression, including binge eating, grazing, depressive symptoms, age, and childhood trauma explained 55% of FA variance, with 7% of variance explained uniquely by childhood trauma.

Conclusions

This study supports that FA is related to childhood trauma and provides a potential explanation for the association of FA with greater psychopathological severity. From a clinical perspective, FA provides an accurate and quick assessment of psychopathological severity and represents an essential complement to the evaluation of eating disorders related to overweight. Future studies should attempt to estimate the impact of childhood trauma on treatment outcomes.

## Introduction

In the last two decades, many neuroimaging and experimental studies using animal models have suggested that addictive processes may be involved in the overconsumption of highly palatable food (i.e., processed calorie-dense foods) [1-2]. These studies rapidly led to the concept of food addiction (FA), which has been operationalized by adapting the diagnostic criteria for substance-related and addictive disorders (SRAD) of the Diagnostic and Statistical Manual of Mental Disorders (DSM-5) [3]. Since then, the Yale Food Addiction Scale has been the measure of choice to assess FA [4-5]. Although FA is not a psychiatric disorder included in the DSM-5, many studies have highlighted its clinical relevance by showing that it was associated with higher BMI and greater psychopathological severity (e.g., higher psychiatric comorbidities, cravings, shape and weight concerns, and psychological distress) and, even more importantly, when co-occurring with binge eating disorder (BED) [6-10]. To account for these findings, Davis proposed that FA might be the most severe form of BED [8]. Reviewing many neurobiological, psychological, and behavioral pieces of evidence, she proposed an (over)eating continuum that presents a dimensional view based on eating severity, degree of compulsiveness, and level of impairment. That continuum starts with homeostatic eating, then passive forms of overeating, and then different forms of loss-of-control eating (i.e., binge eating episodes, BED, and finally FA at the very end of the continuum).

The association between FA and greater psychopathological severity could also be accounted for by more frequent traumatic experiences in the past [11]. The association with childhood cumulative trauma (i.e., an accumulation of several interpersonal trauma during childhood) is well documented, but the associations with specific types of trauma are inconsistent across studies. A preliminary study involving 57,321 women registered nurses showed that physical and sexual abuse were associated with a higher risk of FA [12]. A second study demonstrated, with a sample of 49,408 women, that FA prevalence was twice as high in a severe posttraumatic stress disorder group (6-7 symptoms) compared to a group without trauma history [13]. Recently, a study involving 73 women showed higher childhood cumulative trauma as well as higher physical abuse, emotional abuse, and emotional neglect scores for the FA group than for the no FA group. However, in this study, no differences were found in sexual abuse and physical neglect [14]. Finally, similar results were obtained with bariatric candidates while elevated adverse childhood experience scores were associated with an increased likelihood of FA diagnosis and greater FA severity [15]. To our knowledge, only one study did not find higher childhood cumulative trauma for the FA group compared to the control group [16]. However, the control group from this study showed a sexual abuse score much higher than the cut-off score recommended by Bernstein and Fink [17], suggesting that this group had a more severe trauma history than what would be expected from a control group.

Only one study investigated the association between both FA and binge eating (using the Binge Eating Scale) and childhood trauma [18]. In this study, it was demonstrated that FA and binge eating were positively associated with childhood cumulative trauma, physical abuse, emotional abuse, physical neglect, and emotional neglect but not sexual abuse. Second, childhood cumulative trauma was a significant predictor of FA when controlling for potential confounding variables, including binge eating. Finally, it was demonstrated that the group with co-occurring FA and binge eating had higher childhood cumulative trauma and emotional abuse than groups with FA or binge eating alone and higher physical abuse, physical neglect, and emotional neglect compared to the control group (without FA or binge eating). However, for sexual abuse, no difference was found between groups. The absence of a group difference for sexual abuse as well as the absence of a positive relation between FA and sexual abuse was unexpected, considering previous findings [12]. According to the authors, these unexpected results could be explained by the low rate of sexual abuse experiences reported by their sample [18].

The association between childhood trauma and FA is increasingly evident, but some inconsistencies between specific types of traumas need to be clarified. Moreover, other maladaptive eating behaviors that may be associated with experiences of childhood trauma and FA have been overlooked. Grazing, which is an unstructured, repetitive eating of small amounts of food over a longer time period [19-22], has been suggested to better represent eating behaviors associated with obesity and FA than binge eating [23-24]. However, no study has investigated the association between grazing and childhood trauma, and only one study investigated the association between FA and grazing. This study reported that grazing was a good predictor of FA symptoms [25]. Finally, other types of childhood interpersonal traumas had not been considered. Above all, peer victimization (e.g., teasing, bullying, pushing, and social exclusion) could be associated with FA. Peer victimization is pernicious in its repetitive nature and has been demonstrated to be an important contributor to many psychological problems [26]. In particular, peer victimization has been repeatedly associated with eating disorders [27-30].

The main objective of this study was to examine the relationship between childhood trauma (including peer victimization and abuse and neglect) and maladaptive eating behaviors (FA, binge eating, and grazing). A second objective was to examine whether childhood trauma predicts FA when controlling for binge eating, grazing, and other confounding variables. It was hypothesized that all types of childhood traumas will be positively associated with all maladaptive eating behaviors, but in accordance with the assumption that FA is the more severe form of overeating [8], stronger associations between childhood trauma and FA were expected. Moreover, it was hypothesized that childhood trauma will explain a significant portion of FA variance even when controlling for binge eating, grazing, and other confounding variables.

## Materials and methods

Participants

The sample recruited for this study consisted of 125 adult individuals seeking psychological help for problems related to eating or weight. The sample size estimation established that 125 participants would be sufficient to detect a small effect. Eligibility criteria included (1) being over 18 years old and (2) having overweight or obesity (which was measured either by a self-reported BMI ≥ 25 kg/m^2^ or by endorsing being overweight or obese). Retrospectively, males (nine participants) and non-Caucasians (two participants), considering that they were few, were removed to avoid bias in the analysis. Therefore, the final sample was 114 Caucasian adult women with a mean age of 44.00 years (SD = 13.19). BMI ranged between 25 and 55.10 kg/m^2^ (M = 37.74; SD = 6.68; only 49 participants agreed to provide their weight and height). For diagnosis, 1% met DSM-5 criteria for bulimia nervosa (BN), 30% for binge eating disorder (BED), and 10% were classified as otherwise specified feeding and eating disorder (OSFED; majorly because participants did not reach the frequency or duration criteria for BED), 39% as unspecified feeding and eating disorder (because participants did not report loss of control over eating or an objective large quantity). and 20% did not reach the clinical level for an eating disorder even if they seek psychological help for weight and eating problems.

Procedure

Participants were recruited at the Centre d'Expertise Poids, Image et Alimentation (CEPIA), a multidisciplinary university clinic that offers eating disorder treatment and psychological support for obesity management but no weight loss treatment per se. The recruitment phase lasted from April 2019 to April 2020. All participants who began psychological treatment for problems related to eating or weight were invited to participate in the study. They participated in a DSM-5 diagnosis assessment made by psychologists or trained postgraduate psychology students to screen for eating disorders. In addition, they completed questionnaires on LimeSurvey, an online survey platform hosted on Canadian servers on which all the questionnaires of the study were hosted. Participants were free to provide their weight and height in the questionnaire. With their consent, their data were anonymized and included in a database used for the present study. Data were all collected anonymously. The Laval University Research Ethics Committee approved the study.

Measures

Food Addiction

The Yale Food Addiction Scale 2.0 (YFAS) is a 35-item self-report questionnaire used to assess FA symptoms [5,31]. The YFAS 2.0 covers FA criteria based on the DSM-5 11 diagnostic criteria for SRAD [3]. Items are answered on a seven-point Likert scale, ranging from 0 (never) to 7 (every day). To fulfill a criterion, participants must endorse at least one item related to the criterion. The YFAS 2.0 can be used in two different ways. First, it is possible to assess the presence/absence of the “FA diagnostic” if participants have endorsed at least two criteria and have reported functional impairment or clinical distress. The second method is to assess FA severity by summing up the endorsed criteria (0-11). In the present study, internal consistency was adequate, with a Kuder-Richardson of .81.

Binge Eating

The Binge Eating Scale (BES) is a 16-item self-report questionnaire used to assess symptoms related to behavioral, cognitive, and emotional manifestations of binge eating episodes [32]. For each item, participants are asked to choose, among four statements, the one that best describes their situation. Each item is allocated weight, representing severity (varying between zero and three), and subsequently summed so that total scores vary from 0 to 46: a score of 17 or less indicates the presence of no or few binge-eating episodes, a score of 18 to 26 indicates moderate binge-eating severity or frequency, and a score of 27 or more indicates severe binge eating or high episode frequency and suggests a binge-eating disorder diagnosis. In the present study, internal consistency was adequate with Cronbach's alpha of .86.

Grazing

The Grazing Questionnaire (GQ) is a seven-item self-report questionnaire used to assess behaviors and cognitions specific to grazing with loss of control [21]. Items are answered on a five-point Likert scale ranging from 0 (never) to 4 (always), and all items can be summed for a global score ranging from 0 to 28. The GQ showed high internal consistency (α = .82), good test-retest reliability (r = .67), and convergent validity with other measures of problematic eating behavior such as BES (r = .67) [21]. In the present study, internal consistency was adequate with Cronbach's alpha of .84.

Depressive Symptoms

The Beck Depression Inventory-II (BDI-II) is a 21-item self-report questionnaire used to assess depressive symptoms experienced in the last two weeks [33]. Each symptom is rated on a four-point Likert scale from zero (no suffering) to three (intense suffering). The total score ranges from 0 to 63: a score from 0 to 13 represents normal mood to minimal depressive symptoms, a score from 14 to 19 represents mild to moderate depressive symptoms, a score from 20 to 28 represents moderate depressive symptoms, and a score from 29 to 63 represents severe depressive symptoms. The BDI-II was used because it captures different manifestations (e.g., sadness, guilt, fatigue, motivation) that can be related to distress. In the present study, internal consistency was adequate with Cronbach's alpha of .93.

Body Mass Index (BMI)

BMI was calculated based on a standard procedure (kg/m²) for 49 participants. Thirty-four participants (30%) were measured with metric and weighting scales by a trained research assistant, and 15 participants (13%) provided self-report measures of height and weight.

Peer Victimization

The Adolescent Peer Relations Instrument (APRI) is a 36-item self-report questionnaire divided into two sections of 18 items [34]. The first section measures victimization behaviors committed, and the second section measures victimization behaviors experienced as a victim. Within each section, the three types of victimization behaviors (verbal, physical, and social) are assessed with six items on a six-point Likert scale ranging from 1 (never) to 6 (every day). The questionnaire shows good internal consistency of each of the factors (α = .82 to .92) [34]. For this study, only the second section (being victimized) was used, and three items (one for each type of peer victimization) specifically targeting peer victimization related to weight were added. Therefore, the severity score for each type of peer victimization (verbal, physical, and social) ranges from 7 to 42, and the total score varies from 21 to 126 for global peer victimization. As there was no French version, the APRI was translated using a forward-backward method. The scale was translated from English to French by a bilingual graduate student and then translated from French to English by another bilingual graduate student. Both students and a third independent scholar discussed the final translation until agreement on item formulation was reached. In addition, as the questionnaire assesses peer victimization behaviors committed in the past year at school, the instructions have been adapted for adults to reflect peer victimization behaviors experienced during childhood in general. In the present study, internal consistency was adequate, with Cronbach's alpha ranging from .81 to .96 for the total score and subscales for each type of peer victimization.

Abuse and Neglect

The Childhood Cumulative Trauma Questionnaire (CCTQ) is a self-report questionnaire that aims to measure five types of interpersonal childhood traumas (sexual abuse, physical abuse, psychological abuse, physical neglect, and psychological neglect) [35]. It was created including items derived from previous studies of childhood maltreatment [36-39]. Specifically, for sexual abuse, items aim to describe the abuse according to the relationship with the aggressor (e.g., parent, brother, uncle), type of sexual abuse (e.g., touching, sexual intercourse), and the frequency (total of abuse(s) during childhood). A severity score from 0 to 11 can be computed according to those three items. To cover physical and psychological abuse and neglect, 13 items on a seven-point Likert Scale ranging from 0 (never) to 6 (every day or almost) are used. The severity score ranges from 0 to 30 for physical abuse, from 0 to 18 for psychological abuse, from 0 to 12 for physical neglect, and from 0 to 18 for psychological neglect. Each of the five types of traumas can be summed to produce a score of childhood cumulative abuse and neglect. In the present study, internal consistency was adequate, with Cronbach's alpha of .86 for the total score.

Statistical analysis

The IBM SPSS 23.0 statistical software program (IBM Corp., Armonk, NY) was used. Prior to analyses, all variables' distributions were inspected, and no transformation was needed. Statistical power was evaluated using the GPower software program (http://www.gpower.hhu.de/), and it was determined that the sample size of 114 participants was adequate to detect a small effect (R^2^ of 0.07 or more). Except for BMI, no variable had missing data. First, a Pearson correlation matrix was made with age, BMI, depressive symptoms (BDI), maladaptive eating (YFAS 2.0, BES, and GQ), and childhood trauma (APRI and CCTQ). Second, a two-step hierarchical multiple regression was constructed to predict FA. In the first step, all variables showing a significant correlation with FA were included. In the second step, abuse and neglect and peer victimization total scores were added to determine the unique contribution of abuse and neglect and peer victimization in the explanation of FA when controlling for confounding variables.

## Results

Descriptive statistics

Descriptive statistics are presented in Table 1. Globally, the sample reported a high level of maladaptive eating behaviors and a mean BMI reaching class II obesity (between 35.0 and 39.9). For childhood trauma, physical traumas (peer victimization, abuse, and neglect) were less reported than other types of traumas.

**Table 1:** Descriptive statistics BMI = body mass index

Variables	Means	Standard deviation	Participants (N)
Food addiction (/11)	5.74	3.16	114
Binge eating (/46)	24.13	9.10	114
Grazing (/28)	16.21	5.23	114
Depressive symptoms (/63)	16.06	11.95	114
BMI	37.74	6.68	49
Age	44.00	13.19	114
Victimization (total/126)	25.66	20.80	114
Verbal victimization (/42)	14.67	10.66	114
Physical victimization (/42)	3.45	4.82	114
Social victimization (/42)	7.54	7.78	114
Abuse and neglect (total/89)	13.96	12.80	114
Sexual abuse (/11)	3.02	3.65	114
Physical abuse (/30)	1.07	2.19	114
Psychological abuse (/18)	3.94	5.10	114
Physical neglect (/12)	0.34	0.93	114
Psychological neglect (/18)	5.59	5.24	114

Associations with childhood trauma

Correlations between childhood trauma, maladaptive eating behaviors, depressive symptoms, BMI, and age are presented in Table 2. FA was significantly associated with all trauma measures (r from .18 to .39), except physical neglect (r = .08). The binge eating measure showed significant correlations with all traumas (r from .18 to .26), except social peer victimization, psychological abuse, and physical neglect (r from .03 to .14). Except for sexual abuse, binge eating showed smaller correlations with all traumas than FA. Depressive symptoms showed significant correlations with all traumas (r from .18 to .25), except physical peer victimization, sexual abuse, and physical neglect (r from .09 to .16). Age was significantly and negatively associated with peer victimization measures (r from -.21 to -.39) but not with the abuse and neglect measures (r from -.17 to .11). Finally, for grazing and BMI, no significant correlation was found.

**Table 2:** Pearson's correlations between maladaptive eating behaviors and childhood trauma BMI = body mass index Analyses were also performed including the 11 excluded participants (nine men and two non-Caucasians), which confirmed that the exclusion of these participants did not change the results. N = 49 for BMI * p < 0.05; ** p < 0.01; *** p < 0.001

Variables	Food addiction	Binge eating	Grazing	Depressive symptoms	BMI	Age
Victimization (total)	.34 ***	.20 *	.03	.21 *	.15	-.31 **
Verbal victimization	.30 ***	.18 *	.07	.20 *	.19	-.21 *
Physical victimization	.30 ***	.23 *	.10	.16	-.06	-.25 **
Social victimization	.32 ***	.14	-.07	.20 *	.14	-.39 **
Abuse and neglect (total)	.39 ***	.25 **	.06	.25 **	.09	-.08
Sexual abuse	.18 *	.22 *	.14	.09	.11	.11
Physical abuse	.32 ***	.26 **	.06	.18 *	.06	-.06
Psychological abuse	.33 ***	.14	.04	.25 **	.10	-.08
Physical neglect	.08	.03	-.02	.15	.22	-.04
Psychological neglect	.37 ***	.20 *	-.02	.19 *	.01	-.17
Food addiction	---	.71***	.44***	.33***	.19	-.24**

Variance explanation of FA by childhood trauma

The results of the two-step hierarchical regression model are presented in Table 3. In the first step, binge eating, grazing, depressive symptoms, and age explained 49% of FA variance (using the adj. R^2^). Of all predictors, only binge eating had a significant unique contribution. In the second step, abuse and neglect and peer victimization explained 7% more FA variance. Abuse and neglect and peer victimization both showed a significant unique contribution to the explanation of FA variance but binge eating was still the most important predictor.

**Table 3:** Hierarchical regression models to predict the food addiction score Analyses were also performed including the 11 excluded participants (nine men and two non-Caucasians), which confirmed that the exclusion of these participants did not change the results. * p < 0.05; ** p < 0.01; *** p < 0.001

Variables	Standardized coefficients (β)	t	F	R	R^2^	Adj. R^2^	R^2^ change
Global model (step 1)			28.077***	0.71	0.51	0.49	0.51***
Binge eating	0.640	6.561***					
Grazing	0.074	0.852					
Depressive symptoms	0.090	0.517					
Age	-0.038	-0.506					
Global model (step 2)			24.584***	0.758	0.58	0.56	0.07***
Binge eating	0.580	6.292***					
Grazing	0.095	1.174					
Depressive symptoms	-0.009	-0.126					
Age	-0.005	-0.071					
Victimization	0.190	2.697*					
Abuse and neglect	0.154	2.160*					

## Discussion

The main purpose of this study was to examine the relationship between childhood trauma and maladaptive eating behaviors using a sample of 114 adult women with overweight or obesity who consult for weight and eating difficulties. As hypothesized, significant small to moderate correlations were found between all types of childhood traumas and FA, except for one relation (physical neglect). Regarding binge eating, some correlations with different types of childhood traumas were significant but smaller as compared to FA (except for sexual abuse). With the exception of sexual abuse and physical neglect, the correlation coefficients between childhood traumas and FA were above .30, while the correlation coefficients with binge eating were below .30. In their study, Imperatori et al. also reported stronger associations between childhood trauma and FA than with binge eating [18]. Globally, these results are consistent with previous findings regarding the association between childhood trauma and FA [12,14-15,18] or BED [27-30,40-42]. Grazing and BMI showed no significant correlation with any measure of childhood trauma. BMI has been frequently associated with different types of traumas [43-45] but with a small effect (odds ratio from 1.13 to 1.50). Consistent with these meta-analyses, BMI showed small correlations with some types of traumas, without being significant, probably due to a lack of statistical power. It is possible that the relationship between childhood trauma and BMI may be mostly explained by maladaptive eating behaviors, as weight gain is a frequent consequence of those behaviors. Regarding grazing, to our knowledge, no study has examined the association between grazing and childhood trauma. Considering previous findings suggesting that grazing is a common maladaptive eating behavior associated with FA [20,24-25], we hypothesized that grazing is associated with childhood trauma, but no significant correlation was found. This unexpected result may suggest that grazing is a form of maladaptive eating that is less compulsive than binge eating and FA and, therefore, not specific to people with a trauma history. While the frequency of compulsive grazing is unknown in the general population, a study assessing the frequency of nibbling found that 40% of university students with healthy weight reported nibbling more than half the days in a whole month [46]. In comparison, the prevalence of BED is approximately 1-2% [3], and the prevalence of FA is between 0% and 26% in the general population [47].

A second objective of the study was to examine whether childhood trauma predicts FA when controlling for other maladaptive eating behaviors and confounding variables. As hypothesized, childhood trauma significantly explained FA variance. Previously, Imperatori et al. built a hierarchical regression model to predict FA and found that binge eating, childhood trauma, BMI, and other confounding variables explain 55% of FA variance [18]. However, because childhood trauma was entered in the first step of their hierarchical regression, it is impossible to know the variance (i.e., R^2^ change) associated with the inclusion of childhood trauma. Of all predictors, binge eating, childhood trauma, and BMI were those with a significant unique contribution. They reported standardized coefficients of 0.66 for binge eating and 0.12 for childhood trauma, which is roughly similar to those we obtained (i.e., 0.58 for binge eating, 0.19 for peer victimization, and 0.15 for abuse and neglect). The present results replicated their findings and established a significant contribution of approximately 7% of childhood trauma in the explanation of FA.

Davis proposed an (over)eating continuum that presents a dimensional view of overeating based on severity, degree of compulsiveness, and level of impairment [8]. By referring to the experienced trauma as a marker of severity, our results can support the hypothesis of a continuum of compulsive eating behaviors. In our study, grazing was not discriminant enough to identify people with trauma history and could be placed toward the beginning of the continuum. Because FA showed the strongest associations with trauma history, it might be the best marker of severity and should be placed at the very end of the continuum. Binge eating was less correlated with trauma history than FA but still showed some positive correlations with different types of traumas and should be placed between grazing and FA.

A broad body of literature supports the deleterious long-term effects of peer victimization. For instance, two important reviews defend that peer victimization in childhood and adolescence is a major risk factor for impaired functioning and poor physical and mental health in adults [48-49]. Even though peer victimization behaviors appear less intrusive and severe than sexual and physical abuse, they still represent interpersonal aggression that may be destructive by its repetitive nature. To date, peer victimization has not been considered in studies on childhood trauma and FA. In our study, the association found between peer victimization and FA (r from .30 and .34) suggests that it is likely a contributor as important as other types of traumas.

The current study has limitations to consider. First, BMI was not reported for a significant proportion of participants and could not be included in the hierarchical regression. The absence of a significant correlation between BMI and any measure of childhood trauma may also be a consequence of the high proportion of missing data for BMI. Therefore, results regarding BMI may be interpreted with caution. Second, a self-report scale was used to assess binge eating. This choice was made because a binge eating severity score was necessary for correlations, and BED diagnosis (i.e., a dichotomous variable) would not fit its purpose. Therefore, it is important to distinguish the BED diagnosis from the binge eating severity score used in this study. Finally, this study was only composed of Caucasian women. This choice was made to increase the homogeneity of the sample and the internal validity. Therefore, our results cannot be generalized to males or people with different racial backgrounds. Nevertheless, this study is coherent with the overrepresentation of women in eating disorders and obesity management services. Additionally, the clinical sample in this study makes it impossible to generalize the results to the general population.

## Conclusions

To shed light on the clinical relevance of FA, this study further explored the relation between FA and childhood trauma. Both peer victimization and abuse and neglect showed significant positive correlations with FA and unique contribution to the explanation of FA when controlling for binge eating, grazing, depressive symptoms, and age. These findings provide a potential explanation for the association of FA with greater clinical severity. From a clinical perspective, we argue that FA provides an accurate and quick assessment of psychopathological severity and represents an essential complement to the evaluation of eating disorders related to overweight. The psychopathological severity associated with FA may be the result of biological embedding, which would require long-term treatment. The next step should be to test the impact of childhood trauma on treatment outcomes with participants endorsing FA.
